# A Pilot Study of the Effect of Green Kiwifruit on Human Intestinal Fermentation Measured by Hydrogen and Methane Breath Testing

**DOI:** 10.1089/jmf.2018.4179

**Published:** 2018-12-12

**Authors:** Amanda G.Y. Chen, Marleen S.L. Offereins, Christopher J. Mulder, Christopher M. Frampton, Richard B. Gearry

**Affiliations:** ^1^ Department of Gastroenterology and Medicine, Christchurch Hospital, University of Otago, Christchurch, Christchurch, New Zealand.; ^2^ Department of Medicine, VU Medical Centre, Amsterdam, The Netherlands.

**Keywords:** *breath testing*, *FODMAP*, *intestinal fermentation*, *irritable bowel syndrome*, *kiwifruit*

## Abstract

We investigated the impact of the ingestion of two green kiwifruit (*Actinidia deliciosa* var. Hayward) and one Royal Gala apple on breath hydrogen and methane production in humans. Consumption of two green kiwifruit led to no evidence of carbohydrate malabsorption (0/20), whereas consumption of one apple was associated with carbohydrate malabsorption in 6/20 participants (*P* = .008). There were no significant differences in the area under the curve for hydrogen or methane breath concentrations after consumption of the two fruits. Rates of lactose and fructose breath tests in this cohort were within expected parameters. Green kiwifruit are not associated with clinically significant carbohydrate malabsorption compared with apples in this pilot study.

Functional gastrointestinal disorders (FGDs), including irritable bowel syndrome (IBS), affect over 20% of the population, leading to significant morbidity and healthcare expenditure.^1,2^ The etiology of FGDs is poorly understood, but may include disorders of the brain–gut axis, visceral afferent hypersensitivity, and gastrointestinal dysbiosis.^3^ Different foods may trigger gastrointestinal symptoms such as diarrhea, abdominal bloating, wind, and pain, and most patients identify dietary change as an effective means of controlling symptoms.^4^ For some foods, there is a clear mechanism for the pathogenesis of gastrointestinal symptoms, while the mechanisms of symptom generation are less well understood for other foods. In recent years, FODMAPs (fermentable oligo-, mono-, and disaccharides and polyols), a group of nonabsorbed rapidly fermented carbohydrates, have been described as potent triggers of gastrointestinal symptoms in people with IBS.^5,6^ Low FODMAP diet and the exclusion of other food groups have been subjects of increasing research.^7^ However, there have been few human studies describing whole food digestion, which may exacerbate gut symptoms.

The ingestion of two green kiwifruit (*Actinidia deliciosa* var. Hayward) daily has been shown to improve abdominal symptoms in those with constipation,^8,9^ although the mechanism is poorly understood.^10^ While there has been no signal of increased abdominal bloating or diarrhea in individuals consuming kiwifruit in these clinical studies, other treatments for constipation and gastrointestinal discomfort are associated with such adverse effects.^11^ Hydrogen and methane breath testing has been used in humans routinely to demonstrate excessive intestinal fermentation, which may be associated with gastrointestinal symptoms such as diarrhea and abdominal pain and bloating.^12^ We aimed to compare gastrointestinal fermentation patterns following the ingestion of lactulose, fructose, and lactose (standard test substrates) and the whole foods of two green kiwifruit and one apple (both standard serving sizes) in people with IBS and healthy controls.

Twenty participants (10 IBS and 10 controls) took part in this prospective, nonrandomized, nonblinded, clinical pilot study. This number of participants was discussed with a biostatistician and identified as being appropriate for a pilot study of this type. Cases were identified from outpatient clinics referred by gastroenterologists for diagnostic breath testing and dietary manipulation for the management of IBS. Ten healthy controls were recruited from the community. Inclusion criteria included participant age between 18 and 65 years with a body–mass index of between 18 and 35 kg/m^2^. Those with IBS needed to fulfill the Rome III diagnostic criteria.^13^ Those with other gastrointestinal disorders such as inflammatory bowel disease, diverticulosis, colorectal cancer or polyposis, or bowel resection; other significant health conditions (*e.g.*, diabetes, cardiovascular, or neurological disease); were pregnant or breastfeeding; or had allergies to a test substrate were excluded.

All participants underwent five breath tests over five nonconsecutive days using established and validated methodology.^14,15^ Participants followed a low fermentable diet for 24 hours before each test and were nil by mouth for 10 hours before testing. Breath samples were tested at baseline for H_2_ and CH_4_ concentrations and then every 15 minutes for at least 3 hours after ingestion of each substrate (lactulose 15 g, fructose 35 g, lactose 50 g, one Royal Gala apple [125 g], and two Zespri™ green kiwifruit [190 g]) using a Quitron Microlyser (Quintron Instrument Co., Milwaukee, WI, USA). The primary outcome for the study was the proportion of participants with positive breath hydrogen (rise >10 ppm for greater than two consecutive recordings) or breath methane (rise >20 ppm for greater than two consecutive recordings) tests following the ingestion of each substrate as a measure of clinically significant intestinal fermentation. Secondary outcomes included the area under the curve (AUC) for both H_2_ and CH_4_ production. Ethical approval was given by the University of Otago Human Ethics Committee (H16/008). Written informed consent was obtained from all participants.

Chi-square tests were used to compare the number of positive tests between each group. Friedman's test was used to compare the AUC values from the breath tests. Pairwise comparisons were undertaken using the Wilcoxon signed-rank test when significant differences among the substrates were found (IBM SPSS Statistics, version 23). A value of *P* < .05 was considered statistically significant.

All participants performed the five breath tests except for one participant who did not return for the fructose breath test and was lost to follow-up. There were no significant demographic baseline differences between cases and controls (Table 1). Regarding the primary outcome of the study, there were significantly fewer positive breath tests following the ingestion of two green kiwifruit (0/20) compared with the positive control lactulose (14/20, *P* < .001), fructose (4/20, *P* = .04), lactose (4/20, *P* = .04), and apple (6/20, *P* = .008) (Table 2).

**Table 1.** Characteristics of the Study Groups

*Patient variable*	*Healthy participants*	*IBS patients*	*Total*
Sex, male/female	0/10	0/10	0/20
Age, median (range), years	48 (23–62)	36 (18–54)	40.5 (18–62)
BMI, median (range), kg/m^2^	25.2 (19–31)	29 (21–55)	27.1 (19–55)
Smoking, *n* (%)	1 (10)	0	1 (5)
Alcohol, median, units/week	2 (0.8)	4 (0–10)	3 (0–10)

BMI, body–mass index; IBS, irritable bowel syndrome.

**Table 2.** Breath Test Results of Study Participants

*Patient variable*	*Healthy participants*	*IBS patients*	*Total*
Positive BT after lactulose	7 (70%)	7 (70%)	14 (70%)^a^
Positive BT after fructose	2 (22%)	2 (20%)	4 (21%)^a^
Positive BT after lactose	2 (20%)	2 (20%)	4 (20%)^a^
Positive BT after 2 green kiwifruit	0	0	0
Positive BT after one apple	3 (30%)	3 (30%)	6 (30%)^a^
Kiwifruit BT (H_2_ AUC), mean (SD)^b^	71 (294)	3 (487)	14 (400)^c^
Kiwifruit BT (CH_4_ AUC), mean (SD)^b^	70 (233)	0 (261)	32 (244)
Lactulose BT (H_2_ AUC), mean (SD)^b^	1056 (1226)	2082 (3716)	1569 (2744)^c^
Lactulose BT (CH_4_ AUC), mean (SD)^b^	98 (511)	245 (765)	171 (638)
Fructose BT (H_2_ AUC), mean (SD)^b^	597 (1687)	1163 (2930)	895 (2376)
Fructose BT (CH_4_ AUC), mean (SD)^b^	73 (132)	314 (734)	200 (541)
Lactose BT (H_2_ AUC), mean (SD)^b^	1109 (4635)	341 (526)	725 (3235)
Lactose BT (CH_4_ AUC), mean (SD)^b^	13 (108)	154 (340)	60 (264)
Apple BT (H_2_ AUC), mean (SD)^b^	86 (355)	0 (784)	68 (593)
Apple BT (CH_4_ AUC), mean (SD)^b^	167 (384)	138 (267)	152 (308)

^b^ Measurements in parts per million H_2_ or CH_4._.

^a^ Significant difference between rates of positive breath tests after substrate compared with kiwifruit (*P* < .05).

^c^ *P* < .05 using both Freidman's and Wilcoxon signed-rank tests.

AUC, area under the curve; BT, breath test.

The mean AUC for CH_4_ (32 vs. 171 ppm, *P* = .041) and H_2_ (14 vs. 1569 ppm, *P* = .01) production was significantly lower for green kiwifruit than the positive control lactulose. The CH_4_ and H_2_ AUCs for kiwifruit were nonsignificantly lower than for fructose, lactose, and apple. Rates of fructose and lactose-positive breath tests were consistent with population rates. There were no differences in the AUCs between the control and IBS groups (*P* > .05).

This pilot study has demonstrated no evidence of clinically significant carbohydrate malabsorption following the ingestion of two green kiwifruit in a cohort of healthy controls and adults with IBS. Rates of breath test positivity for fructose, lactose, and the positive control lactulose in this mixed cohort were within expected population ranges.^16^ H_2_ and CH_4_ breath testing is used to diagnose individuals with carbohydrate malabsorption that may trigger gut symptoms.^15,17^ Consumption of two green kiwifruit daily has been shown in clinical trials to reduce constipation and to improve abdominal discomfort^8,9^ and, in the present study, this has not been associated with clinically significant carbohydrate malabsorption. This suggests that kiwifruit may be less likely to induce luminal distension and abnormal gas handling in the colon, both of which may lead to abdominal bloating, flatus, and discomfort.^18^

Green kiwifruit are low in free fructose, which may be poorly absorbed and rapidly fermented in some individuals.^19^ There are similar amounts of fructose and glucose (4.68 and 4.13 g/100 g of edible flesh, respectively) in green kiwifruit, facilitating cotransport across the apical membrane into the enterocyte.^20,21^ Additionally, there are negligible amounts of other nonabsorbed carbohydrates that could act as substrates for colonic fermentation.^22^

This pilot study, surprisingly, demonstrated clinically significant increases in breath H_2_ or CH_4_ (consistent with malabsorption of the substrate using accepted criteria) in 30% of participants following the ingestion of one apple. Apples contain 5.9 g of fructose and 2.4 g of glucose. While this amount of free fructose would seem unlikely to lead to clinically significant malabsorption, free fructose, in addition to sorbitol, may make apples more likely to trigger gut symptoms in some people with IBS. Other potential explanations for the findings relate to differences in the fruit matrix and digestion in the small intestine. Fiber content is unlikely to lead to significant differences in colonic fermentation given that the fiber content for two green kiwifruit and one Royal Gala apple is 5.8 and 2.6 g, respectively.^19^

This pilot study is not without limitations. First, the number of participants is small, all female, and the findings need to be replicated in a larger group of participants. Furthermore, while there were trends, no significant differences were seen between the kiwifruit and other substrate AUCs (with the exception of the positive control lactulose), which may have supported these findings. The duration of breath testing following the ingestion of substrates may not have been long enough to capture significant colonic fermentation that could have occurred after the 3-hour window. However, the final breath test recordings at 3 hours were approaching baseline in all individuals and it is likely that such delays will have affected breath test recordings for both fruits equally. It is also possible that malabsorption of other components of the kiwifruit could have led to symptoms. Other measures of colonic fermentation such as measurement of short-chain fatty acids in the fecal stream were not performed in this pilot study and may lead to a deeper understanding of the fate of kiwifruit digesta in the colon in humans.^23^ In conclusion, the consumption of two green kiwifruit was not associated with evidence of clinically significant colonic fermentation in the 3 hours after consumption in a mixed population of IBS and healthy participants.
